# ECG-surv: A deep learning-based model to predict time to 1-year mortality from 12-lead electrocardiogram

**DOI:** 10.1016/j.bj.2024.100732

**Published:** 2024-05-01

**Authors:** Ching-Heng Lin, Zhi-Yong Liu, Jung-Sheng Chen, Yang C. Fann, Ming-Shien Wen, Chang-Fu Kuo

**Affiliations:** a Center for Artificial Intelligence in Medicine, Chang Gung Memorial Hospital, Taoyuan, Taiwan; b Bachelor Program in Artificial Intelligence, Chang Gung University, Taoyuan, Taiwan; c Division of Intramural Research, National Institute of Neurological Disorders and Stroke, National Institutes of Health, Bethesda, MD, United States; d Division of Cardiology, Chang Gung Memorial Hospital, Taoyuan, Taiwan; e School of Medicine, Chang Gung University, Taoyuan, Taiwan; f Division of Rheumatology, Allergy and Immunology, Chang Gung Memorial Hospital, Taoyuan, Taiwan

**Keywords:** Survival prediction, Electrocardiography, Censored data, Deep neural network, Deep learning

## Abstract

**Background:**

Electrocardiogram (ECG) abnormalities have demonstrated potential as prognostic indicators of patient survival. However, the traditional statistical approach is constrained by structured data input, limiting its ability to fully leverage the predictive value of ECG data in prognostic modeling.

**Methods:**

This study aims to introduce and evaluate a deep-learning model to simultaneously handle censored data and unstructured ECG data for survival analysis. We herein introduce a novel deep neural network called ECG-surv, which includes a feature extraction neural network and a time-to-event analysis neural network. The proposed model is specifically designed to predict the time to 1-year mortality by extracting and analyzing unique features from 12-lead ECG data. ECG-surv was evaluated using both an independent test set and an external set, which were collected using different ECG devices.

**Results:**

The performance of ECG-surv surpassed that of the Cox proportional model, which included demographics and ECG waveform parameters, in predicting 1-year all-cause mortality, with a significantly higher concordance index (C-index) in ECG-surv than in the Cox model using both the independent test set (0.860 [95% CI: 0.859–0.861] vs. 0.796 [95% CI: 0.791–0.800]) and the external test set (0.813 [95% CI: 0.807–0.814] vs. 0.764 [95% CI: 0.755–0.770]). ECG-surv also demonstrated exceptional predictive ability for cardiovascular death (C-index of 0.891 [95% CI: 0.890–0.893]), outperforming the Framingham risk Cox model (C-index of 0.734 [95% CI: 0.715–0.752]).

**Conclusion:**

ECG-surv effectively utilized unstructured ECG data in a survival analysis. It outperformed traditional statistical approaches in predicting 1-year all-cause mortality and cardiovascular death, which makes it a valuable tool for predicting patient survival.

## Highlights

• ECG-surv is a deep neural network capable of effectively analyzing unstructured ECG data while simultaneously addressing censoring issues.
• Comprehensive evaluations have indicated that ECG-surv is robust and surpasses conventional statistical approaches and clinical risk scores in accurately predicting short- and long-term mortality risks.
• ECG-surv can serve as a tool for condensing complex ECG data into a single measure with predictive capabilities.

## 1 Introduction

Prediction of mortality risk plays a crucial role in identifying patients at heightened risk for death and informing clinical decision-making. Accurately predicting patient survival serves as the cornerstone for developing personalized treatment plans. By accurately estimating a patient's survival probability, clinicians can make informed decisions about each individual's most appropriate treatment options. This personalized approach allows for tailoring treatments to the specific needs and characteristics of the patient, optimizing their chances of survival and minimizing potential side effects. Prediction rules are often based on demographic and clinical variables derived from large observational studies. Although the Framingham risk score (FRS) [1] is a widely used and accepted tool for risk stratification, its accuracy and applicability are limited because of its exclusion of certain well-known risk factors [2]. In addition, despite the proven value of the electrocardiogram (ECG) in cardiovascular risk prediction [3,4], the FRS (like most clinical prediction algorithms developed from traditional statistical methods) fails to incorporate unstructured data such as ECG data. Traditional statistical methods operate under the assumption that data is structured and conforms to a tabular format with distinct rows and columns. Furthermore, these methods are built upon assumptions regarding the data distribution and variable interactions, assumptions that are often not applicable to unstructured data. Incorporating ECG data may improve mortality risk prediction [[5], [6], [7], [8]].

Many clinical trials and other studies have demonstrated that deep learning algorithms can extract subtle ECG features that are challenging to identify by human reading, thereby improving disease diagnosis or prediction [[9], [10], [11]]. Recent studies have highlighted the potential for deep neural networks (DNNs) to predict mortality from a 12-lead ECG [4,12,13]. However, typical DNN models do not explicitly incorporate censored data, unlike Cox proportional hazards models [[14], [15], [16], [17]]. Recent advances in deep learning have introduced various architectures to overcome this limitation, such as DeepSurv [18] and DeepHit [19]. Nevertheless, these algorithms remain confined to the use of structured data.

We designed a deep learning model that combines the strengths of the ResNet [20] and DeepHit architectures. ResNet has demonstrated its efficiency in extracting ECG features in previous studies [[21], [22], [23], [24]], making it ideal for analyzing unstructured ECG data. In contrast, DeepHit is a non-proportional discrete-time neural network model that considers the joint distribution of survival time and events without assuming a specific stochastic process. Thus, this model architecture was able to handle unstructured ECG data and tackle challenges related to censoring simultaneously. Our predictive model, called ECG-surv, was trained from more than 4 million ECG examinations. Furthermore, we conducted both internal and external validation using data from an independent hospital to ensure the robustness of the model. In addition, we carried out a comprehensive performance comparison between ECG-surv and other Cox models built from ECG parameters and FRS factors. The present study explored the potential for utilizing the output of ECG-surv as input for subsequent models, expanding the range of applications for our approach.

## 2 Methods

### 2.1 Study populations and setting

**Fig. 1:**
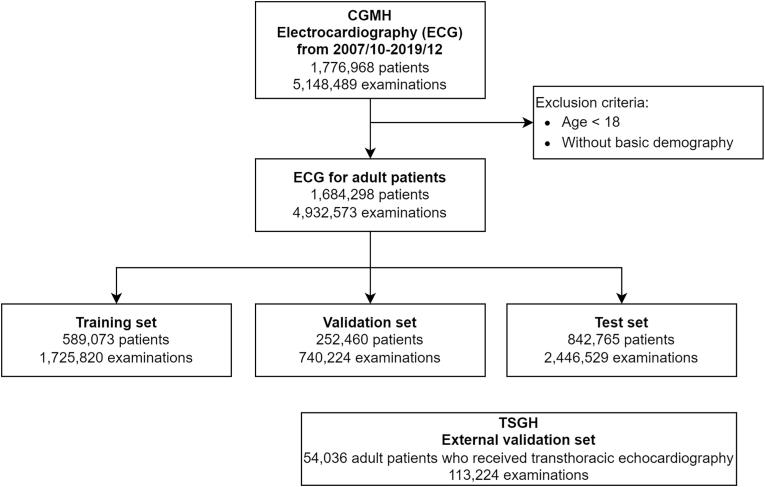
Summary of the data used in the study. The data from the CGMH were split into training, validation, and test sets. The data from the TSGH were used as an external validation set. Note that the CGMH ECGs were acquired using a MAC 5000 ECG machine (GE Healthcare, Chicago, IL, USA) and the TSGH ECGs were acquired using a Philips 12-lead ECG machine. Abbreviation: CGMH, Chang Gung Memorial Hospital; TSGH, Tri-Service General Hospital; ECG, electrocardiogram.

Two study populations were used to train and evaluate our deep learning models [Fig. 1]. The first study population included patients who underwent standard 12-lead ECG examination at Chang Gung Memorial Hospital (CGMH) from October 2007 to December 2019. The ECG data were linked to the Chang Gung Research Database, which included the electronic health records of all patients who visited one of the following seven hospitals: Keelung, Taipei, Linko (headquarters), Taoyuan, Yunlin, Chiayi, or Kaohsiung. These hospitals are situated in varied settings, including urban, suburban, and rural areas, with two major medical centers being the Linkou and Kaohsiung branches. This approach facilitates the comprehensive collection of diverse patient information. In total, 4,932,573 ECGs from 1,684,298 patients at CGMH were eligible for analysis. The patients were randomly assigned to a training set, validation set, and test set using stratified random sampling by birth year and sex, and each patient had an equal probability of selection without replacement. Patients were not shared among the training, validation, and test sets. We additionally collected 113,224 ECGs from 54,036 adult patients at Tri-Service General Hospital (TSGH) for independent external validation of the predictive models.

The primary outcome of this study was 1-year all-cause mortality, and the secondary outcome was 1-year cardiovascular death. In the case of a single ECG scenario, the index date was defined as the time of that particular ECG examination. In an individual instance, the index date was set as the time of the most recent ECG examination for that individual. Cardiovascular diseases (CVDs) were identified using the International Classification of Diseases, Tenth Revision, Clinical Modification (ICD-10-CM) codes I00–I99 and ICD-9-CM codes 390–459. The patients’ data were linked to the National Registry of Deaths provided by the Ministry of Health and Welfare in Taiwan.

The study was approved by the Institutional Review Boards of Chang Gung Medical Foundation and TSGH. The requirement for informed consent was waived because of the retrospective nature of the study and secondary analysis of existing data. All aspects of the investigation adhered to relevant clinical research guidelines and regulations.

### 2.2 ECG acquisition

The CGMH ECG dataset contained resting standard 12-lead ECGs with 10 s voltage–time traces that were acquired at a sampling rate of 500 Hz using a MAC 5000 ECG machine (GE Healthcare, Chicago, IL, USA) and digitally stored in a MUSE™ Cardiology Information System (GE Healthcare). Then, each ECG was converted into a 12 × 5000 matrix for input into the model. The ECGs from the TSGH dataset were recorded using a Philips system, following the same sample rate and recording time settings as in the CGMH dataset. No additional padding of shorter duration was applied to either dataset.

### 2.3 ECG-surv DEVELOPMENT

We developed the ECG-surv model to handle both survival time and censored data. ECG-surv is a one-dimensional convolutional neural network that builds upon the ResNet [20] and DeepHit [19] architectures. We employed a one-dimensional ResNet-18 neural network to extract features from patients’ 12-lead ECGs. Then, these extracted features were fed into the DeepHit model to estimate the risk of mortality. DeepHit is a nonproportional discrete-time neural network model that estimates the joint distribution of survival time and events without making any assumptions about the underlying stochastic process. It accounts for the possibility that the relationship between covariates and risks varies over time.

**Fig. 2:**
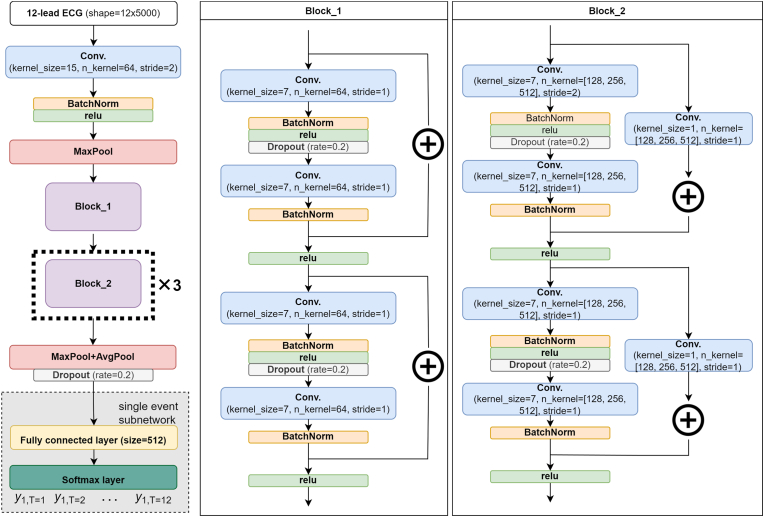
Architecture of the ECG-surv model, which concatenates a ResNet-18 model and a DeepHit model. Abbreviation: ECG: electrocardiogram; Conv: convolutional layer; Block_1 and Block_2 represent two types of residual block.

The overall model architecture is illustrated in [Fig. 2]. The ECG-surv architecture contains a convolution layer followed by a residual block of static channel (Block_1) and three residual blocks of incremental channel (Block_2). The output of each convolutional layer was followed by batch normalization for distribution normalization and fed into a rectified linear unit activation function layer and a dropout layer with a rate of 0.2 for model regularization. The output of the last block was fed into hybrid (max and average) pooling [25]. Finally, the output of the hybrid pooling layer was sent to a DeepHit single-event subnetwork with a single softmax layer as the final output layer to estimate the probability mass function (PMF) of the survival distribution. The final output layer consisted of 12 neurons representing 12-month time bins of follow-up time. The models were constructed using PyTorch 1.7.1 and Python 3.8.5.

### 2.4 ECG-COX model development

We developed the ECG-Cox model to compare ECG-surv with traditional statistical Cox models that included demographics and ECG waveform parameters. The ECG-Cox model is a multivariate Cox proportional hazards model that includes age at the time of ECG recording, sex, and eight ECG-specific variables (heart rate, PR interval, QRS duration, QT interval, QTc interval, P axis, R axis, and T axis) extracted from 1,725,820 ECGs in the CGMH training set. These variables are important predictors of mortality [[26], [27], [28]] and are available in both the MUSE™ (GE Healthcare) and Philips ECG information systems.

### 2.5 Framingham Cox model development

Two Cox proportional hazards models were developed to compare ECG-surv with the Framingham scoring system: one model used Framingham risk factors (Framingham risk factor Cox model) and the other used the FRS (FRS Cox model). The Framingham risk factor Cox model was fit with sex, age at the time of ECG recording, total cholesterol, high-density lipoprotein cholesterol, diastolic, and systolic blood pressure, diabetes, and smoking status [1]. We also built a univariate Cox proportional hazards model to directly incorporate the FRS as the FRS Cox model. We selected patients with a record of their smoking status in the CGMH test sets for training and testing of the two Cox models. The training set included 53,342 records from 36,768 patients, and the test set included 76,045 records from 46,887 patients.

### 2.6 Expanding the application of ECG-surv

**Fig. 3:**
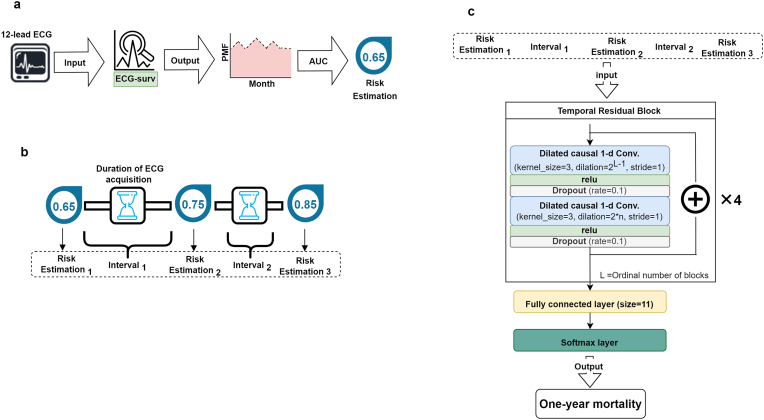
Overview of ECG-surv risk estimation. **a** The AUC of the PMF value output from ECG-surv was considered a risk estimation of an ECG. **b** A series of risk estimations and corresponding time intervals were considered sequential data. **c** A temporal convolutional network model deals with the risk-interval sequential data for 1-year all-cause mortality prediction. Abbreviation: ECG: electrocardiogram; AUC: area under the curve; PMF: probability mass function.

To expand the use of ECG-surv, we utilized its output as an estimate for cardiac health. DeepHit, a part of ECG-surv, parameterizes the PMF and combines the log-likelihood to calculate the probability of survival in discrete time intervals [19]. In the context of survival analysis, the PMF refers to the probability distribution function of a discrete random variable that is used to model the time until an event occurs (e.g., death or censoring). The PMF values at discrete time points can be plotted as a curve. This curve is considered a representation of a heart condition estimate from an ECG by ECG-surv. To obtain a numerical value for the ECG estimate, the composite trapezoidal rule is employed to calculate the area under the PMF curve [Fig. 3a].

We proposed two scenarios to illustrate the use of ECG-surv estimates. The first scenario utilized the estimate as an input parameter for other prediction models. We built a multivariate Cox proportional hazards model that included the ECG-surv estimate, the patient's age at the time of ECG examination, and the patient's sex. The model was trained using the CGMH training set. The other scenario utilized multiple ECG-surv estimates for predicting 1-year mortality. ECG-surv takes a single 12-lead ECG as the input. To take sequential temporal information into account and fully utilize multiple ECG data, we developed a predictive model that processes multiple ECG-surv estimates based on a temporal convolutional network (TCN) [29]. A TCN is specifically designed to capture long-term temporal dependencies in sequential data by employing convolutional layers to extract distinctive features from the input sequences. These multiple estimates from ECG-surv can be considered a sequence of time-stamped heart checkup tests; thus, we sequenced the test results and time intervals between two consecutive results. In other words, each patient's ECG estimation is expressed as a long sequence of ECG-surv risk estimations in which the duration between two consecutive estimations appears in the sequence [Fig. 3b]. In our dataset, each patient had an average of 4.5 ECGs in the CGMH cohort and 3.0 ECGs in the TSGH cohort.

A TCN model was designed to deal with the sequence for 1-year mortality forecasting [Fig. 3c]. This model comprised four temporal residual blocks and one fully connected layer as the output layer. The temporal residual block consisted of two layers of dilated causal convolutions with a dilation size of 2^L−1^ (in which L is the ordinal number of blocks), a rectified linear unit activation function layer, and a dropout layer with a rate of 0.1 between the convolutions. The input to the TCN model was a sequence with a length ranging from 2 to 23; namely, the model input required at least 2 ECG estimation results with 1 time interval and up to 12 estimation results with 11 time intervals. Sequences shorter than 23 were padded with zeros, and longer sequences were truncated to 23. The ECG estimation result was the survival probability calculated by ECG-surv, and the time interval unit was 1 week. The output of this model was a binary label indicating whether the patient survived or died within one year after the last ECG examination date. The models were constructed using PyTorch 1.7.1 and Python 3.8.5.

### 2.7 Deep learning model training and validation procedure

We trained, validated, and tested ECG-surv in the CGMH cohort, which included 1,725,820 ECGs from 589,073 patients in the training set (35% of entire CGMH dataset), 740,224 ECGs from 252,460 patients in the validation set (15% of entire CGMH dataset), and 842,765 ECGs from 842,765 patients in the test set (50% of entire CGMH dataset). We performed additional external validation in the form of a second study population from TSGH containing 74,400 ECGs from 18,843 patients. ECG-surv was optimized using the Adam method of stochastic gradient descent with a learning rate of 1e-4 and batch size of 512 with 5 epochs. The loss function was adopted from DeepHit, which consists of the log-likelihood of the joint distribution of the first hitting time and event as well as a cause-specific ranking loss function [19]. Model hyperparameters were chosen by grid search hyperparameter optimization in the validation set.

To prepare the data for TCN model training, we excluded patients with only one ECG examination in both the CGMH and TSGH cohorts. There were 273,194 patients left in the training set, 116,700 patients left in the validation set, 362,833 patients in the test set, and 18,842 patients in the external validation set. The median number of ECG examinations per patient was three and two in the CGMH and TSGH cohorts, respectively. The TCN model was optimized by binary cross-entropy using the Adam method of stochastic gradient descent with a learning rate of 5e-4 and batch size of 512 with 10 epochs. Model hyperparameters were chosen by grid search hyperparameter optimization in the validation set.

All training was performed on an NVIDIA DGX-1 platform with two V100 GPUs and 32 GB of RAM per GPU (NVIDIA Corporation, Santa Clara, CA, USA).

### 2.8 Evaluation of model prediction performance

Performance measures and model behavior were evaluated using only the test and external validation datasets. The time-dependent C-index served as the performance metric for the ECG-surv, ECG-Cox, and Framingham risk factor/FRS Cox models. The C-index is a generalization of the area under the receiver operating characteristic curve (AUROC) that takes censored data into account. It measures the concordance between the ordering of actual survival times and the predicted risk ordering by the model [30]. Similar to the AUROC, a C-index of 1 represents the best model prediction and a C-index of 0.5 represents a random prediction. The discriminative ability of the predictive model with multiple ECG-surv inputs was evaluated using the AUROC. A bootstrapping technique was employed to generate a 95% confidence interval (CI).

### 2.9 Waveform analysis

To assess the behavior of ECG-surv, we used a saliency map [31] to objectively determine the salient regions of a particular lead contributing to the 1-year mortality risk estimation. Saliency maps contribute to the interpretation of electrocardiograms by providing visualizations of the important features learned by deep learning model. The saliency map was generated by passing the ECG data through the model to update the gradients, followed by backtracking to the input layer to compute the derivative of the output layer concerning each value in the input ECG signal. We calculated the median ECG waveforms for each lead with a total time step of 600 ms of 100,000 randomly selected ECGs from the CGMH test set and the entire TSGH external validation set. We overlaid the median ECG waveforms along with the ECG saliency maps to depict areas of the ECG in which changes had the greatest influence on risk estimates. In addition, we randomly selected 50,000 ECGs with predicted low risk and 50,000 ECGs with predicted high risk from the CGMH test set and the entire TSGH external validation set for median ECG waveform analysis.

## 3 Results

### 3.1 Patient characteristics

We used original ECG signal data from patients who underwent one or more standard 12-lead ECG examinations at CGMH or TSGH. Information on the patients’ baseline age, sex, medical history, blood pressure, cholesterol, and smoking status was collected from the electronic medical records at the time of ECG recording. The mean age of the overall study population was 60.5 ± 16.7 years in the CGMH cohort and 66.3 ± 16.1 years in the TSGH cohort. [Table 1] shows the characteristics of the training, validation, test, and external validation sets. There were no significant differences between the training, validation, and test sets in the CGMH cohort (absolute standardized mean difference <0.2), but age was significantly different between the CGMH test dataset and the TSGH dataset. The TSGH set has a slightly higher mean age, indicating a potentially older population. In the CGMH dataset, a substantial proportion of the population are non-smokers, yet over a third of patients exhibit smoking behavior. In the CGMH and TSGH cohorts, 517,705 (10.5%) and 11,835 (10.5%) ECGs were recorded within 1 year prior to death, respectively.

**Table 1:** Baseline characteristics and comorbidities of CGMH set across training, validation, and test sets and TSGH set.

Characteristic	Training (*n* = 1,725,820)	Validation (*n* = 740,224)	Test (*n* = 2,466,529)	ASMD^a^^,^^b^	TSGH (*n* = 113,224)	ASMD^a^^,^^c^
Age, years	60.5 ± 16.8	60.5 ± 16.7	60.5 ± 16.7	0.002	66.3 ± 16.1	0.353
Age GROUP, YEARS				0.036		0.330
<40	217,614 (12.6)	92,896 (12.5)	313,156 (12.7)		7497 (6.6)	
40–49	213,118 (12.3)	92,635 (12.5)	303,883 (12.3)		8474 (7.5)	
50–59	307,625 (17.8)	131,582 (17.8)	440,055 (17.8)		16,974 (15.0)	
60–69	336,073 (19.5)	142,810 (19.3)	477,763 (19.4)		25,198 (22.3)	
70–79	298,589 (17.3)	128,047 (17.3)	424,481 (17.2)		21,225 (18.7)	
≥80	352,801 (20.4)	152,254 (20.6)	507,191 (20.6)		33,856 (29.9)	
Sex				0.002		0.008
Female	819,737 (47.5)	351,259 (47.5)	1,168,790 (47.4)		53,212 (47.0)	
Male	906,083 (52.5)	388,965 (52.5)	1,297,739 (52.6)		60,012 (53.0)	
Medical history						
Diabetes mellitus	461,092 (26.7)	195,587 (26.4)	658,305 (26.7)	0.007	–	–
HYPERLIPIDEMIA	74,624 (4.3)	30,778 (4.2)	103,472 (4.2)	0.008	–	–
Renal disease	276,277 (16.0)	117,573 (15.9)	385,446 (15.6)	0.010	–	–
Hypertension	854,239 (49.5)	363,376 (49.1)	1,213,928 (49.2)	0.008	–	–
Coronary artery disease	361,703 (21.0)	156,433 (21.1)	515,626 (20.9)	0.006	–	–
Myocardial infarction	136,957 (7.9)	58,106 (7.8)	193,437 (7.8)	0.003	–	–
No medical history	687,909 (39.9)	297,697 (40.2)	988,726 (40.1)	0.007	–	–
Framingham risk factor						
Total cholesterol, *n*	53,342	22,520	76,045		–	–
	168.7 ± 48.4	167.3 ± 48.2	167.9 ± 47.7	0.030	–	–
LDL cholesterol, *n*	53,108	22,434	75,719		–	–
	102.6 ± 44.8	102.1 ± 45.0	102.4 ± 44.4	0.012	–	–
HDL cholesterol, *n*	53,428	22,539	76,189			
	39.2 ± 13.5	38.8 ± 13.5	39.0 ± 13.3	0.037		
Diastolic blood pressure, *n*	53,428	22,539	76,189		–	–
	76.0 ± 10.8	76.0 ± 10.6	76.1 ± 10.8	0.003	–	–
Systolic blood pressure, *n*	53,428	22,539	76,189		–	–
	129.9 ± 18.1	129.9 ± 18.0	130.0 ± 18.1	0.004	–	–
Smoking status, *n*	53,428	22,539	76,189		–	–
No	35,378 (66.2)	14,806 (65.7)	49,895 (65.5)	0.015	–	–
Yes	18,050 (33.8)	7733 (34.3)	26,294 (34.5)		–	–
Death						
Within 3 months	102,314 (5.9)	43,364 (5.9)	145,872 (5.9)	0.003	7749 (6.8)	0.038
Within 6 months	135,775 (7.9)	57,250 (7.7)	193,345 (7.8)	0.005	9574 (8.5)	0.023
Within 9 months	160,320 (9.3)	67,814 (9.2)	228,181 (9.3)	0.004	10,783 (9.5)	0.009
Within 1 YEAR	181,907 (10.5)	76,933 (10.4)	258,865 (10.5)	0.004	11,835 (10.5)	0.001

*n*, number of electrocardiogram records. Data are presented as *n* (%) or mean ± standard deviation.

a ASMD > 0.2 was considered a sign of important imbalance.

b The balance of covariates between training and validation, training and test, and validation and test datasets for CGMH were measured using the ASMD. The largest value of the ASMD was reported.

c The balance of covariates between the test dataset of CGMH and the TSGH dataset. Abbreviation: CGMH: Chang Gung Memorial Hospital; TSGH: Tri-Service General Hospital; ASMD: absolute standardized mean difference; LDL: low-density lipoprotein; HDL: high-density lipoprotein.

### 3.2 ECG-surv PERFORMANCE

**Fig. 4:**
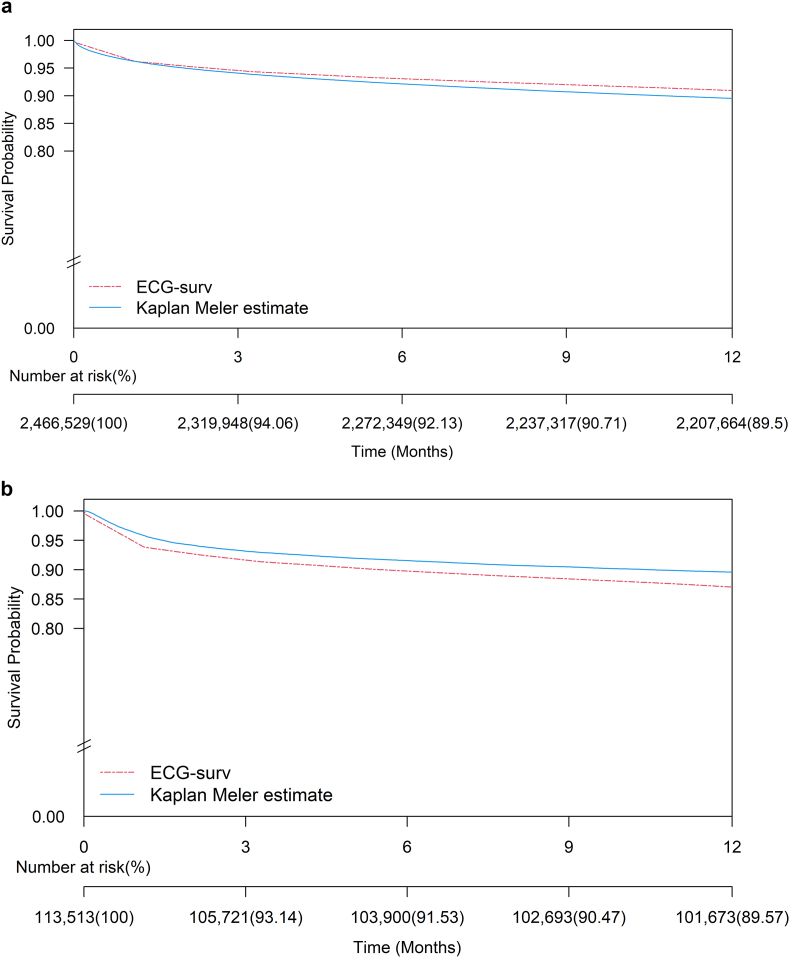
Kaplan–Meier curve (blue line) and ECG-surv estimate (red line) of electrocardiogram records in the **a** CGMH test dataset and **b** TSGH dataset. Abbreviation: CGMH: Chang Gung Memorial Hospital; TSGH: Tri-Service General Hospital.

The performance of ECG-surv was evaluated using the test set and external validation set. In the CGMH test set, ECG-surv demonstrated moderate discrimination for all-cause mortality as indicated by a C-index of 0.860 (95% CI: 0.859–0.861). Similarly, in the TSGH external validation set, ECG-surv exhibited a C-index of 0.813 (95% CI: 0.807–0.814) for all-cause mortality prediction. The model consistently performed well across different ECG devices; the GE Healthcare device was used for the test set, and the Philips device was used for the external validation set. We compared the ECG-surv estimate with the Kaplan–Meier survival curve for both the CGMH test set and TSGH external validation set [Fig. 4]. The ECG-surv estimate was the mean of the risk estimates over all ECGs in the test and external validation sets. Although the log-rank tests of both estimates were statistically significant (*p* < 0.05), the mean absolute difference between the ECG-surv and Kaplan–Meier estimates was small in the CGMH test set (1%) and external validation set (2%). These differences were not considered clinically significant. We further evaluated the performance of ECG-surv in CVD death, and the C-index was 0.891 (95% CI: 0.890–0.893). The CVD death characteristics in the CGMH cohort are presented in [Supplementary Table 1].

### 3.3 Comparisons with adjusted cox model including demographics and ECG waveform parameters

**Fig. 5:**
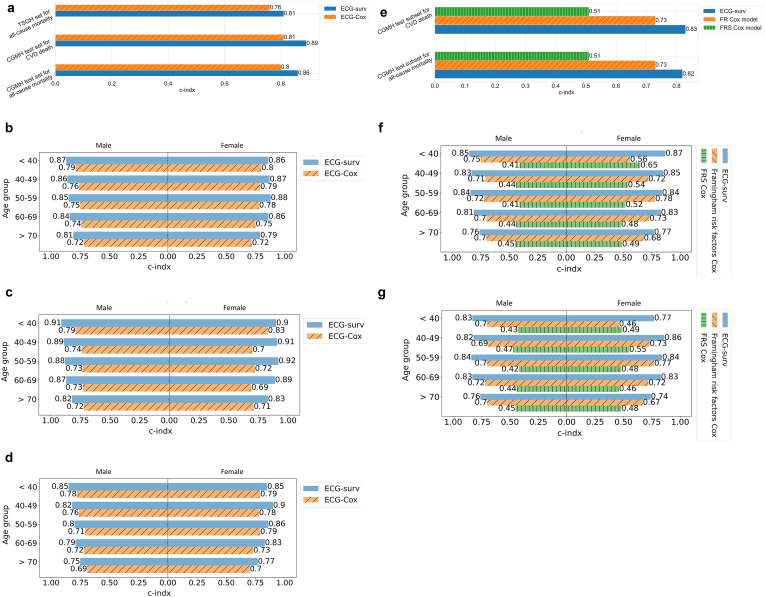
Comparisons of performance of ECG-surv and different Cox models. **a** C-index performance of ECG-surv and ECG-Cox tested on the CGMH test set for all-cause mortality, CGMH test set for CVD death, and TSGH external validation set for all-cause mortality. **b** Age-stratified comparison of ECG-surv and ECG-Cox between the sexes in the CGMH test set for all-cause mortality. **c** Age-stratified comparison of ECG-surv and ECG-Cox between the sexes in the CGMH test set for CVD death. **d** Age-stratified comparison of ECG-surv and ECG-Cox between the sexes in the TSGH set for all-cause mortality. **e** C-index performance of ECG-surv, Framingham risk factor Cox model, and FRS Cox model tested on the CGMH test set for all-cause mortality and for CVD death. **f** Age-stratified comparison of ECG-surv, Framingham risk factor Cox model, and FRS Cox model between the sexes in the CGMH test set for all-cause mortality. **g** Age-stratified comparison of ECG-surv, Framingham risk factor Cox model, and FRS Cox model between the sexes in the CGMH test set for CVD death. Abbreviation: CGMH: Chang Gung Memorial Hospital; TSGH: Tri-Service General Hospital; CVD: cardiovascular disease; Framingham risk factor Cox model Framingham risk factor Cox model; FRS Cox model: Framingham risk score Cox model; C-index: concordance index.

We fitted a multivariate Cox proportional hazards model in the CGMH training set (i.e., ECG-Cox) as a benchmark model. ECG-Cox was built from structured data including patient demographics and ECG waveform parameters. The C-index of ECG-Cox was 0.796 (95% CI: 0.791–0.800) for all-cause mortality prediction in the CGMH test set, 0.809 (95% CI: 0.806–0.811) for CVD death prediction in the test set, and 0.764 (95% CI: 0.755–0.770) for all-cause mortality prediction in the external validation set. ECG-surv exhibited significantly better performance (*p* < 0.05) than ECG-Cox [Fig. 5a]. We also performed an age-stratified comparison of ECG-surv and ECG-Cox in different age and sex groups. The performances of both models decreased with increasing age in men, and the highest performance was in women aged 50–59 years. A dramatic decrease in the performance of ECG-surv was observed in the ≥70-year-old age group for all-cause mortality prediction in the TSGH set (the C-index decreased by about 0.1 for both sexes).

### 3.4 Comparisons with Framingham cox models

To compare the performance of the ECG-surv and Framingham Cox models, we identified patients with complete Framingham risk parameters at the time of ECG examination (about 3% of all CGMH data) [Table 1]. The average FRS for the subset was 7.9. The C-index for the Framingham risk factor model was 0.734 (95% CI: 0.715–0.752) for both all-cause mortality and CVD death prediction. Notably, that for the FRS Cox model was 0.513 (95% CI: 0.492–0.536) for both all-cause mortality and CVD death prediction. The additional performance evaluation results of the FRS Cox and Framingham risk factor Cox models, constructed by incorporating additional variables including the number of ECGs and interventions, are presented in [Supplementary Table 2]. ECG-surv outperformed both Cox models, even in age-stratified comparisons.

### 3.5 Extended utilization of ECG-surv

The survival distribution estimated by ECG-surv can be conceptualized as an estimation of the cardiac condition. Consequently, the estimated risk provided by ECG-surv was incorporated as an input parameter in a Cox model for predicting death. [Table 2] presents the Cox model tested on 1-, 3-, 5-, and 10-year follow-up times after ECG examination. Compared to ECG-surv, the model slightly improved by a C-index of 0.01 for both 1-year all-cause mortality and CVD death prediction. For long-term survival prediction, the model had a C-index of 0.838 (95% CI: 0.835–0.840), 0.829 (95% CI: 0.824–0.831), and 0.818 (95% CI: 0.813–0.821) for all-cause mortality follow-up over 3, 5, and 10 years, respectively. For CVD prediction, the model had a C-index of 0.821 (95% CI: 0.818–0.823), 0.811 (95% CI: 0.808–0.814), and 0.800 (95% CI: 0.795–0.805) for 3-, 5-, and 10-year follow-up, respectively.

**Table 2:** C-index performance of multivariate Cox model built with age, sex, and ECG-surv estimate tested on 1-, 3-, 5-, and 10-year follow-up times.

	1-year follow-up (95% CI)	3-year follow-up (95% CI)	5-year follow-up (95% CI)	10-year follow-up (95% CI)
CGMH test subset for all-cause mortality	0.860 (0.859–0.861)	0.838 (0.835–0.840)	0.829 (0.824–0.831)	0.818 (0.813–0.821)
CGMH test subset for cardiovascular disease death	0.891 (0.890–0.893)	0.821 (0.818–0.823)	0.811 (0.808–0.814)	0.800 (0.795–0.805)

Abbreviation: C-index: concordance index; CGMH: Chang Gung Memorial Hospital; CI: confidence interval.

**Fig. 6:**
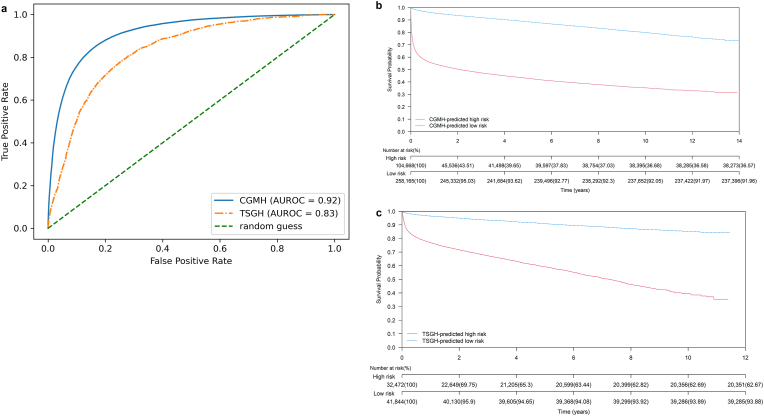
Performance of 1-year all-cause mortality prediction model using multiple ECG-surv estimates for each patient. **a** Receiver operating characteristic curves of the predictive model for the CGMH test set (blue line) and TSGH external validation set (orange line). **b** Long-term Kaplan–Meier survival curves for predicted 1-year all-cause mortality groups of the CGMH test set. **c** Long-term Kaplan–Meier survival curves for predicted 1-year all-cause mortality groups of the TSGH set. Abbreviation: CGMH: Chang Gung Memorial Hospital; TSGH: Tri-Service General Hospital; AUROC: area under the receiver operating characteristics curve.

Expanding on the concept of utilizing ECG-surv estimates as outcomes from cardiac assessments, we developed a binary TCN model that integrates multiple ECG-surv estimates to predict 1-year all-cause mortality for each patient. The model achieved an AUROC of 0.92 in the CGMH test set and an AUROC of 0.83 in the TSGH external validation set [Fig. 6a].

Although this TCN model focused on 1-year mortality prediction, we further estimate the model's ability to perform long-term survival prediction. We performed age- and sex-weighted Kaplan–Meier survival analysis with the available follow-up data stratified by the model prediction. For the group predicted to have 1-year mortality, the median survival times (interquartile ranges) were 0.76 (0.05–2.19) years and 1.82 (0.96–3.33) years in the CGMH test set and TSGH external validation set, respectively. Conversely, for the group predicted to be alive after 1 year, the median survival times were 3.02 (1.70–5.53) years and 3.05 (1.76–4.89) years in the CGMH test set and TSGH external validation set, respectively. The hazard ratio for 1-year mortality was 10.14 (95% CI: 9.97–10.31) and 6.83 (95% CI: 6.53–7.13) in the predicted death group within the CGMH test set and TSGH external validation set, respectively [Fig. 6b and c].

### 3.6 Model behavior

We used saliency maps to explain the predictions made by our deep learning model, which is commonly perceived as a black box, thus providing insights into the reasoning behind the model's decisions. In both the CGMH and TSGH cohorts, the saliency maps illustrated that the P wave and surrounding regions were highlighted in conjunction with the most significant effect in ECG-surv risk estimation [Supplementary Fig. 1]. The median 12-lead ECG waveform analysis demonstrated that patients with high estimated mortality risk tended to have a flatter P wave and ST segment and a lower R wave [Supplementary Fig. 2].

## 4 Discussion

This study evaluated the use of ECG-surv, a DNN based on a 12-lead ECG tracing, to predict all-cause mortality. ECG-surv can simultaneously handle censored data and unstructured ECG data (such as ECG tracings). It significantly outperformed various traditional statistical Cox models that were constructed using ECG waveform measurements or Framingham risk factors. The ECG is widely used in clinical practice due to its diverse range of applications. By incorporating ECG-surv, healthcare professionals can potentially identify high-risk mortality groups through a single examination. This AI model shows promise for integration into ambulatory ECG devices, enhancing their functionality and clinical utility. Consequently, ECG tracing can be effectively employed as a screening tool to identify patients at short- and long-term risks for mortality.

Our results substantiate earlier research that utilized DNNs to predict an event risk from an ECG [32,33]. The 1-year survival estimate produced by ECG-surv demonstrated good discrimination with a C-index of 0.860, even with ECG data obtained using a different device in an external validation set. The generalizability of medical AI models holds crucial importance as it determines the ability of the model to consistently and accurately perform across various real-world environments. In our study, we examined two different brands of electrocardiogram signals due to their significant market presence. The experimental findings displayed a high level of generalizability, thus ensuring successful implementation in patient care and potentially enhancing accuracy and efficacy in real-world scenarios.

The ECG parameters demonstrate more excellent predictive capability in comparison to the risk factors employed in the FRS. This can be observed by contrasting the ECG-surv and ECG-cox models, which solely utilize ECG signals, with the FRS series models. The FRS incorporates various factors such as basic demographics, results from physical examinations, invasive blood tests, and medical history, making the data collection process complex. On the other hand, collecting data from a single non-invasive ECG examination is simpler. The ECG-based models provide immediate risk assessment results following the examination, making it a faster and safer assessment tool. However, the FRS still maintains a significant advantage in terms of calculation convenience. When comparing ECG-surv with ECG-cox, the former demonstrates superior performance. This can be attributed to the deep learning model's ability to extract more representative features compared to the features designed by humans. It suggests that there may be numerous subtle ECG features that are associated with the risk of mortality, which remain unknown to us. Notably, ECG-surv achieved a higher C-index in the CVD death group than in the all-cause mortality group (0.891 vs. 0.860, respectively). This is consistent with the established relationship between ECG abnormalities and CVD [34,35]. ECG-surv significantly outperformed various traditional statistical Cox models built with ECG waveform measurements or Framingham risk factors. This underscores the power of deep learning and the unstructured 12-lead ECG in conferring superior predictive power compared to the complex risk factors used in traditional scoring systems.

Several studies have investigated the use of deep learning techniques to analyze ECGs for predicting the risk of mortality. In a previous study, a DNN was assessed for its ability to directly predict 1-year all-cause mortality using 12-lead ECG data. This model demonstrated a strong predictive capability, AUROC of 0.876 for 1-year mortality [4]. Similar approaches using ECG to predict 1-year [36] and 5-year mortality [37] both achieve an AUC of 0.83. Furthermore, Lima et al. discovered a correlation between electrocardiographic age that surpasses chronological age by over 8 years and increased mortality [38]. Kondo et al. used a two-dimensional CNN to evaluate ECG images for cardiac disease severity in patients, achieving a 77.3% accuracy rate [39]. Tsai et al. proposed a model architecture similar to ours to handle unstructured and censored ECG data [40]. Building upon these foundations, our research introduces models that not only incorporate survival time, thereby enhancing flexibility and performance, but also integrate multiple sources of information, mimicking the comprehensive approach employed by clinicians. Unlike traditional models that may rely on a singular type of data, our methodology reflects the multifaceted nature of clinical decision-making. In practice, physicians base their prognoses on a holistic view of the patient's medical history, employing a blend of various diagnostic tools and insights. Our models emulate this by allowing for the integration of diverse inputs, akin to clinician judgment, thereby providing a more robust tool for clinical application. This innovative approach offers a dynamic risk assessment over time, accommodating varying follow-up periods and analyzing the influence of multiple covariates on survival time. The ability to handle censored data and incorporate multiple inputs represents a significant step forward, enabling a more nuanced understanding of mortality risk factors and aligning closely with the complexity of real-world clinical environments.

ECG-surv is not only a prediction model; its output can be considered an estimate for a heart condition based on ECG data. ECG-surv reduces complex ECG data into a single measure with predictive power. Our performance analysis of the Cox model built with age, sex, and the ECG-surv estimate indicated that the ECG-surv estimate could be a valid predictor of patient survival. This result is unsurprising, given the well-researched correlation between sex, age, and mortality risk. By including these variables in the prediction model, their predictive power can be directly utilized. Notably, Raghunath et al. proposed a more fundamental integration by concatenating age and sex variables into the deep learning model architecture [4]. Another application of the ECG-surv estimate is using it as a sequence of heart checkup results of a prediction model. We developed a TCN predictive model that digests a sequence of multiple ECG-surv estimates and intervals of consecutive examinations to predict 1-year mortality. Through this TCN model, the ECG-surv risk estimates incorporated with temporal information could be utilized to predict 1-year mortality with an AUROC of 0.92. Although this study focused on 1-year mortality risk prediction, our survival analysis results indicate that ECG-surv can add substantial prognostic information to long-term mortality.

There are several limitations to this study. First, it was conducted at an academic medical center, where patients typically present with more complex medical conditions. Although the CGMH dataset also incorporated ECGs from health check centers (4.4%) and outpatient clinics (30.8%), our results may not be broadly applicable to populations with relatively good health. Second, our data collection encompassed urban, suburban, and rural regions spanning from north to south in Taiwan, thereby constraining the generalizability of our findings to other ethnicities.

## 5 Conclusions

Our research introduces ECG-surv, a DNN that predicts the time to 1-year mortality by learning unique features in 12-lead ECG data and addressing censored data issues. It outperformed Cox proportional hazards models in predicting 1-year all-cause mortality and achieved a superior C-index. Moreover, compared to the FRS, ECG-surv was more effective for predicting cardiovascular death. ECG-surv leverages the power of unstructured ECG data in survival analysis, providing a valuable tool for predicting patient survival in the clinical setting. With its highly accurate predictions, this model could potentially facilitate timely interventions and enhance patient care.

## Declaration of competing interest

The authors declare that they have no known competing financial interests or personal relationships that may have influenced the work reported in this paper.
