# Supplementary material for: ECG-surv: A deep learning-based model to predict time to 1-year mortality from 12-lead electrocardiogram
Source: Biomed J. 2024 May 1;48(1):100732. doi: 10.1016/j.bj.2024.100732 (PMC11751416; doi:10.1016/j.bj.2024.100732)
Supplement: Multimedia component 1 [file mmc1.docx]

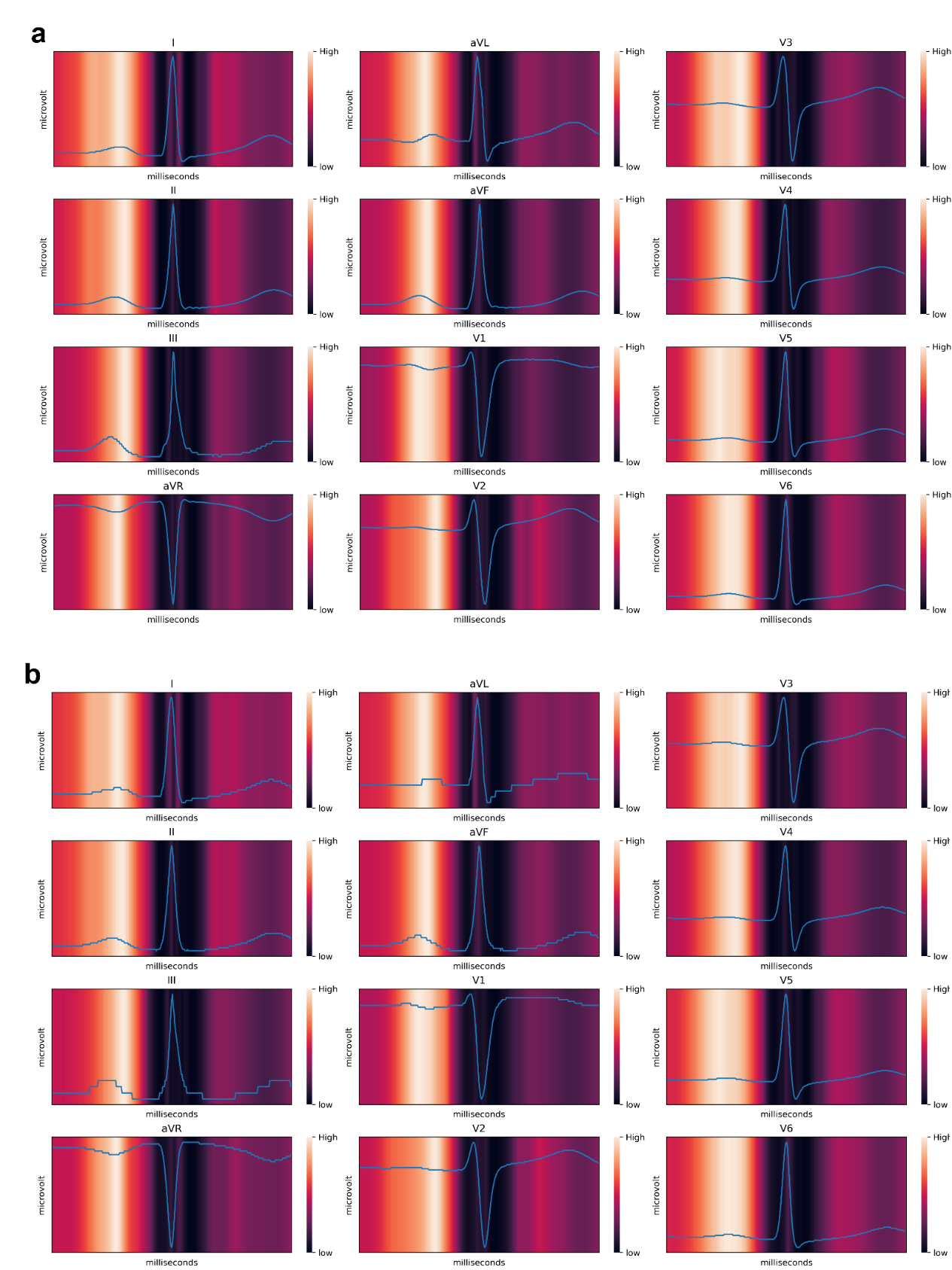


**Supplementary Fig. 1.** Saliency maps of ECG-surv presenting regions of the ECG waveforms that had greater influence on 1-year all-cause mortality risk prediction. a Saliency maps of 100,000 randomly selected ECGs from CGMH test dataset. b Saliency maps of TSGH external validation dataset. CGMH, Chang Gung Memorial Hospital; TSGH, Tri-Service General Hospital; ECG, electrocardiogram.


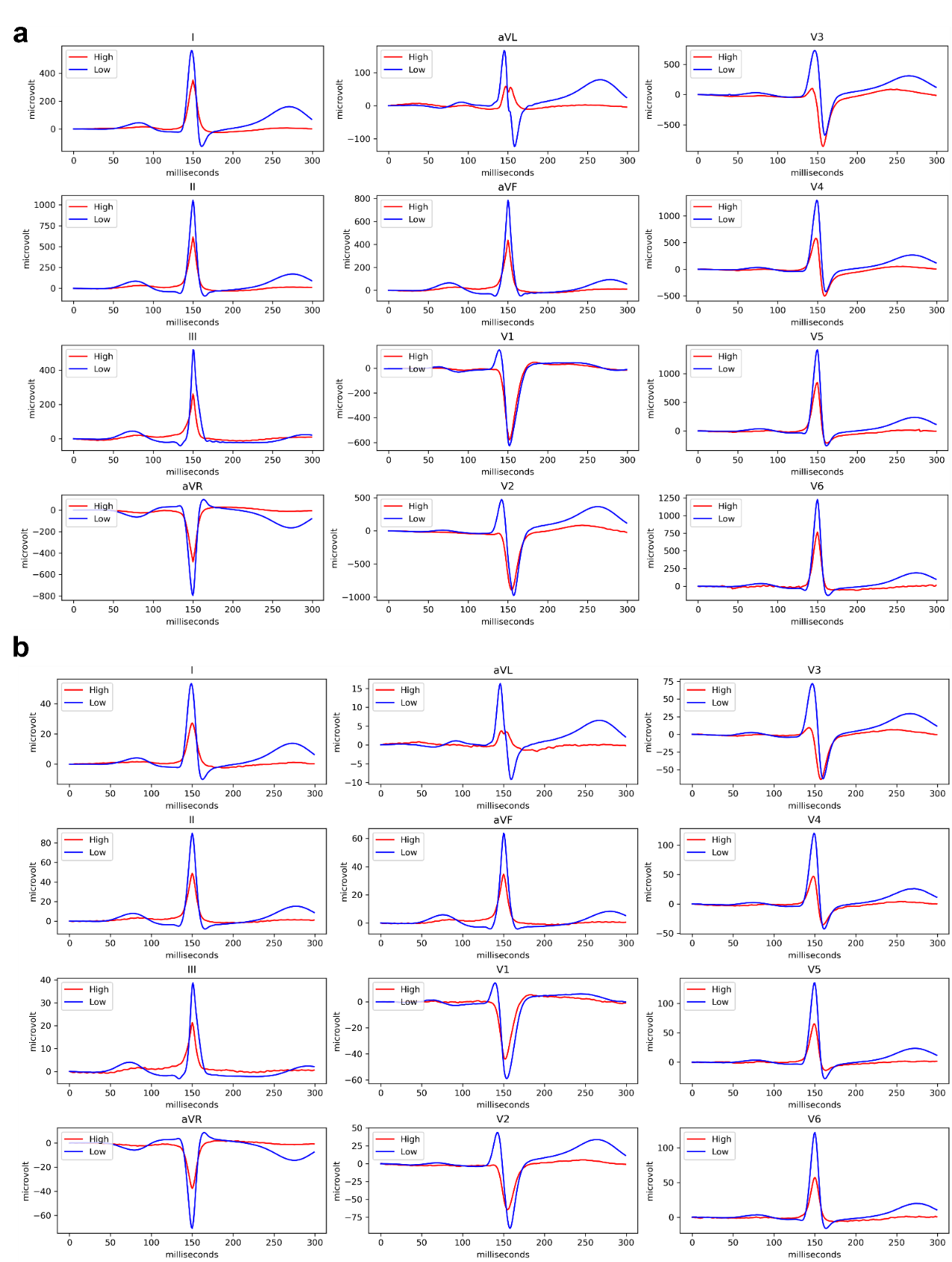


**Supplementary Fig. 2.** Median ECG waveforms of low predicted mortality risk ECG (blue line) compared with high predicted all-cause mortality risk ECG (red line). a Random samples of 50,000 ECGs with predicted low risk and 50,000 ECGs with predicted high risk from CGMH test set. b ECGs from TSGH external validation set. CGMH, Chang Gung Memorial Hospital; TSGH, Tri-Service General Hospital; ECG, electrocardiogram.

**Supplementary Table 1.** Baseline characteristics and comorbidities of cardiovascular diseases (CVD) death patients in Chang Gung Memorial Hospital (CGMH) test set.

| Characteristic | CGMH-CVD death  (*n* = 55,281) |
| --- | --- |
| Age years, mean ± SD | 75.0±13.0 |
| Age groups, *n* (%) |  |
| < 40 | 778 (1.4) |
| 40–49 | 1,683 (3.0) |
| 50–59 | 4,056 (7.3) |
| 60–69 | 8,103 (14.7) |
| 70–79 | 13,159 (23.8) |
| 80 + | 27,502 (49.7) |
| Sex, *n* (%) |  |
| Female | 23,799 (43.1) |
| Male | 31,482 (56.9) |
| Medical history, (%) |  |
| Diabetes mellitus | 20,644 (37.3) |
| Hyperlipidaemia | 2,070 (3.7) |
| Renal disease | 18,119 (32.8) |
| Hypertension | 34,924 (63.2) |
| Coronary artery disease | 23,458 (42.4) |
| Myocardial infarction | 16,674 (30.2) |
| No medical history | 9,027 (16.3) |
| Death, *n* (%) |  |
| Within 3 months | 32,046 (58.0) |
| Within 6 months | 41,506 (75.1) |
| Within 9 months | 48,616 (87.9) |
| Within 1 years | 55,281 (100.0) |

*n* = number of ECG

**Supplementary Table 2.** C-index performance of FRS Cox and Framingham risk factor Cox models built with additional variables. These models were tested on a 1-year mortality using the Chang Gung Memorial Hospital (CGMH) test dataset.

| **Model** | **Additional variable** | | | | **1-year follow-up**  **(95% CI)** |
| --- | --- | --- | --- | --- | --- |
|  | #ECGs | Feq. | CCI | Rx |  |
| FRS Cox | ○ | ○ |  |  | 0.602 (0.582-0.624) |
| FRS Cox |  |  | ○ |  | 0.555 (0.535-0.577) |
| FRS Cox |  |  |  | ○ | 0.640 (0.620-0.661) |
| Framingham risk factor Cox | ○ | ○ |  |  | 0.738 (0.719-0.757) |
| Framingham risk factor Cox |  |  | ○ |  | 0.733 (0.714-0.752) |
| Framingham risk factor Cox |  |  |  | ○ | 0.756 (0.738-0.774) |

# ECGs, number of previous-year ECG examinations conducted;

Feq., whether the patient underwent more than two ECG examinations within the past three months;

CCI, Charlson Comorbidity Index;

Rx, prescription records of Statin, Aspirin, ACEI, ARB, and beta-blocker from the medication data of the previous year;
